# Impact of rice paddy agriculture on habitat usage of migratory shorebirds at the rice paddy scale in Korea

**DOI:** 10.1038/s41598-022-09708-6

**Published:** 2022-04-06

**Authors:** Seung-Hye Choi, Green Choi, Hyung-Kyu Nam

**Affiliations:** 1 Education Support Division, Seoul Science Center, Seoul, 01792 Republic of Korea; 2 GREEN Together Institute, Seocheon, 33646 Republic of Korea; 3 National Institute of Biological Resources, Incheon, 22689 Republic of Korea

**Keywords:** Ecology, Zoology

## Abstract

Approximately 58 shorebird species, including endangered and threatened species, use various habitats while traveling on their long-distance migratory routes in the East Asian-Australasian Flyway (EAAF). Coastal rice paddies in midwestern Korea, which are located in the EAAF, serve as inland wetlands and provide important stopover sites for long-distance migratory shorebirds. We studied how shorebird population density is affected across periods, time since habitat formation, and field type, at the rice field scale. The shorebirds most frequently observed in rice paddies were, in order, black-tailed godwits (*Limosa limosa*), common greenshanks (*Tringa nebularia*), and wood sandpipers (*T. glareola*). Black-tailed godwits and wood sandpipers were affected by time since formation, field type, and water level, whereas field type affected common greenshanks. We propose that (1) flooding time, (2) shallow water level, (3) harrowed field type, and (4) 5–7 days of management intervals at paddy fields are important factors influencing shorebird species density, although all the factors did not influence common greenshank density. We propose that environmental characteristics derived from field management in rice paddies influence habitat use by migratory shorebirds. These factors need to be considered to systematically protect and manage shorebirds that use rice paddies as stopovers during their migration events.

## Introduction

Suitable habitat availability during long-range migrations is essential for shorebirds, including endangered and threatened species, around the world^1^. Most migratory shorebirds have suffered population decline due to sudden changes in habitat^2^. In particular, population loss has clearly occurred along the East Asian–Australasian Flyway (EAAF)^3,4^. For example, the abundance of 11 shorebird species using the EAAF declined by 70–97% in 1999–2015^5^. On this flyway, the major habitats for shorebirds are tidal flats and other coastal wetlands, which undergo constant land-use change^6,7^. Some studies have been conducted on habitats used by migratory shorebirds in regions characterized by land-use change in coastal wetlands, including rice paddies^8–10^, coastal pastures^11^, and shrimp farms^12^. When shorebirds use a habitat that has been altered by land-use change, they must choose how to use the habitat and how frequently^12^.

Rice paddies constitute 15% of global wetlands by area^13^ and provide a valuable habitat for bird species^14,15^. Paddies located along coasts provide important stopover sites for shorebirds, whose populations are decreasing; this has been observed in Europe^16^, North America^17^, and Asia^18,19^. Due to the loss of natural habitats, in some regions, rice paddies provide essential habitats for certain species, including those requiring special conservation attention^20,21^. They also commonly function as alternative habitats to complement natural wetlands^22^. Ultimately, management of rice paddies is an important factor in the conservation of shorebirds^15^.

Rice paddies form a temporary wetland^14^. During the rice-growing season (including preparation for seeding), field management alters the paddy considerably, compared with its surface during the nongrowing season (post-harvest until preparation for seeding the next year). Thus, paddy management during the growing season is crucial to its use as a habitat by numerous waterbird species^23,24^. Despite this, most research on paddy use by waterbirds and on methods of paddy management has been conducted during the nongrowing season^15,25–28^. In particular, there has been little research on paddy use by shorebirds during the rice-growing season^29^.

In Korea, rice paddies are flooded in 5–6 months of the year (from late April to September), a period that coincides with the breeding and migration seasons of waterbirds, such as herons and shorebirds^19,30^. Rice cultivation involves the following stages: plowing and harrowing with flooding (late April and May), planting (late May and early June), growing (June to September), and harvesting (mainly October). The rice plants reach their full height of about 1 m in August. Water levels in rice fields are maintained at 1–20 cm throughout the rice-growing season (From late April to September), after which the fields are allowed to dry out prior to harvesting. Rice fields remain dry from October to early April^31^.

The rice paddies in the midwestern region of Korea are located along the EAAF^19^. This flyway encompasses a vast area, including the tip of Siberia and the southern parts of Alaska, Southeast Asia, Australia, and New Zealand^30^. The area is used by 5 million shorebirds belonging to 58 species as a stopover to replenish energy^32^ on the way to their winter range. Shorebirds breed at high latitudes, and the paddies located within this flyway in midwestern Korea form a bottleneck where huge populations of shorebirds gather during migration^33^.

In the current study, we investigated the use of rice paddies by migrating shorebirds in midwestern Korea during the rice-growing season and investigated how the density of migratory shorebirds was affected by field management. We predicted that potential food sources (rice seeds and benthic organisms) and the population density of shorebirds would differ according to the field type and the time since habitat formation. Our results can facilitate better paddy management practices in coastal inland areas, which function as stopover sites for shorebirds, to assist in the conservation of these species.

## Results

### Habitat-use characteristics

**Figure 1:**
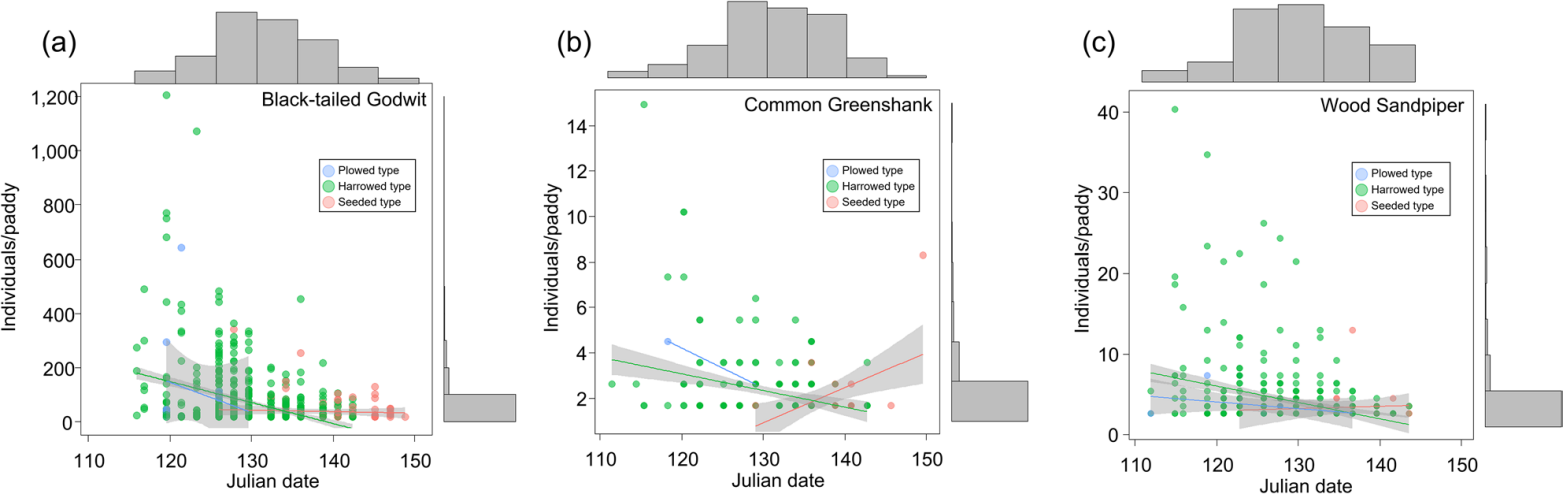
Scatterplot of Julian date and bird density (individuals/paddy) of shorebird species. Each variable represents marginal histograms and 95% confidence interval. The Julian date was divided according to field type (plowed, harrowed, and seeded field): (**a**) Black-tailed Godwit; (**b**) Common Greenshank; (**c**) Wood Sandpiper.

The shorebirds began to visit once the paddies were partially filled with water, which occurred simultaneously with plowing, prior to harrowing and seeding. A total 7,852 bird sightings belonging to 15 shorebird species were observed in the paddies in the study area. Black-tailed godwits (*Limosa limosa*), common greenshanks (*Tringa nebularia*), and wood sandpipers (*Tringa glareola*)—three species that were frequently observed in the study area—were selected as species of interest^[^^18,28^ and were first observed in the Julian dates of 114, 111, and 111, respectively (Fig. 1). During survey periods, we checked a total of 13,660 paddies (plowed = 2,197; harrowed = 6,083; seeded = 5,380). The numbers of paddies used by the three species of shorebirds and the densities (average number of individuals per paddy) were as follows: black-tailed godwits, 809 paddies and 2.53 individuals/paddy (range: 0–1,198 individuals/paddy); common greenshanks, 250 paddies and 0.03 individuals/paddy (0–15 individuals/paddy); and wood sandpipers, 465 paddies and 0.09 individuals/paddy (0–41 individuals/paddy) (Table 1).

**Table 1:** Number of shorebirds observed in each rice paddy field type.

Species	Field type	N	Mean	SE	Min	Max
Black-tailed Godwit	Plowed	2,197	0.53	0.32	0	624
	Harrowed	6,083	5.13	0.49	0	1,198
	Seeded	5,380	0.41	0.10	0	328
Common Greenshank	Plowed	2,197	0.003	0.002	0	4
	Harrowed	6,083	0.06	0.01	0	15
	Seeded	5,380	0.01	0.002	0	8
Wood Sandpiper	Plowed	2,197	0.02	0.005	0	6
	Harrowed	6,083	0.19	0.02	0	41
	Seeded	5,380	0.01	0.003	0	12

N = total number of paddies during survey periods, SE = standard error, Min = minimum number of birds per paddy, and Max = maximum number of birds per paddy.

**Figure 2:**
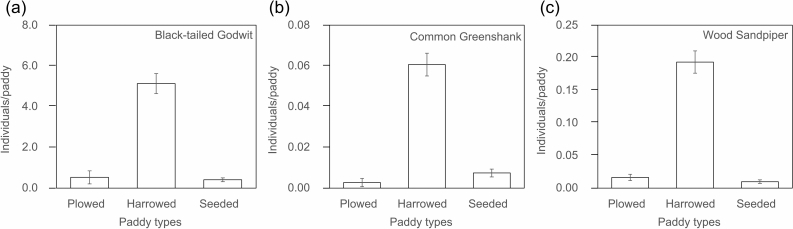
Barplots with error bars showing the mean density (individuals/paddy) of shorebird species in different types of rice fields. The total number of samples from plowed, harrowed, and seeded field types for the survey periods were 2,197, 6,083, and 5,380, respectively. Error bars indicate the standard error: (**a**) Black-tailed Godwit; (**b**) Common Greenshank; (**c**) Wood Sandpiper.

Period and field type had significant effects on the densities of three shorebird species, including black-tailed godwits, common greenshanks, and wood sandpipers. Black-tailed godwits, common greenshanks, and wood sandpipers increased sharply after first observation and were highly frequently observed on Julian dates of 125, 136, and 122, respectively (Fig. 1). In terms of field type, all three species showed higher observation frequencies and total number of individuals at harrowed paddies than at the other two field types (Table 1 and Fig. 2).

**Figure 3:**
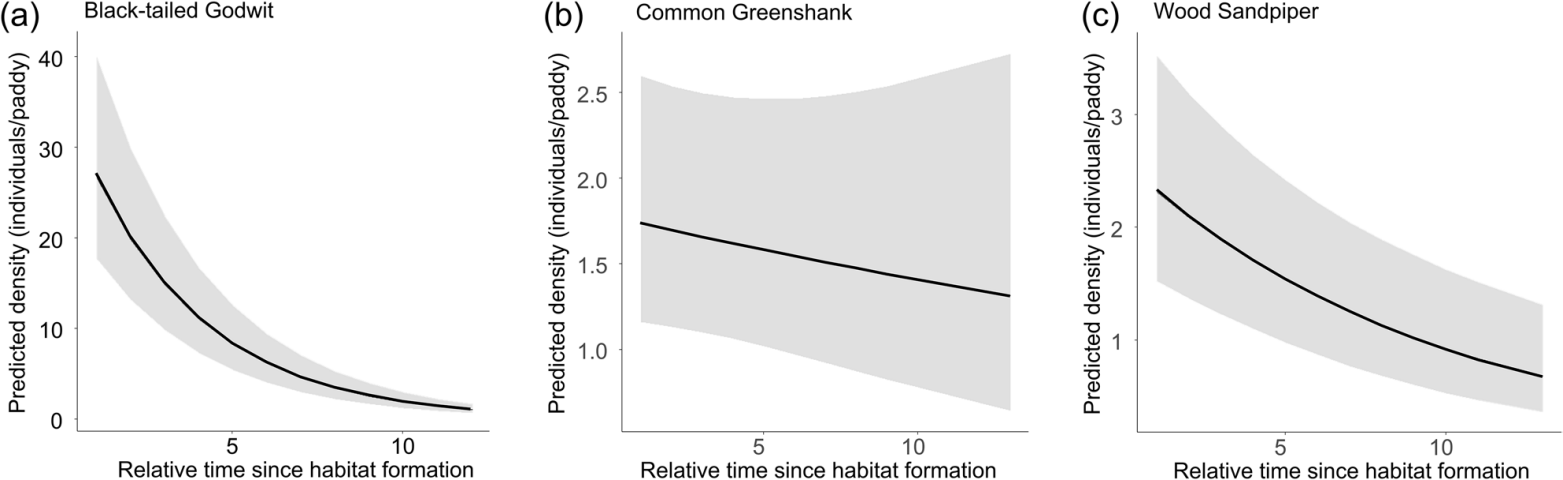
Line graphs showing the predicted density (individuals/paddy) of shorebirds based on relative time since habitat formation. The predicted density was estimated using a generalised linear mixed model with predicted density (individuals/paddy), as the response variable (sample size: black-tailed godwit = 809; common greenshank = 250; wood sandpiper = 465), field type (plowed, harrowed, or seeded) and time since habitat formation as explanatory variables, and survey time and individual paddy as stochastic variables. Lines show the mean predicted density, and the grey-shaded area shows the 95% confidence interval. Each unit on the x-axis represents relative time since habitat formation in 2–3-day intervals: (**a**) Black-tailed Godwit; (**b**) Common Greenshank; (**c**) Wood Sandpiper.

Black-tailed godwits were observed in plowed, harrowed, and seeded paddies that had been maintained in that state for a maximum of 14, 22, and 20 days, respectively; common greenshanks were observed in paddies maintained for 2, 18, and 10 days, respectively; and wood sandpipers, were observed in paddies maintained for 16, 22, and 14 days, respectively. There was a significant effect of time since habitat formation on both black-tailed godwits and wood sandpipers (Table 2), with both species showing their highest densities immediately after field management (black-tailed godwit: mean = 26.98 density; confidence interval [CI] = 18.10–30.23 density; wood sandpiper: mean = 2.83 density; CI = 2.12–3.77 density). Large numbers of birds used the paddies in their early stages after formation, and there was a gradual decreasing trend in use over time (Fig. 3). Field type or time since formation had no significant effects on density of common greenshanks (Table 2). However, there was a significant period and time since formation interaction effect on three shorebird species (Table 2). In addition, large numbers of birds used the paddies in their early stages following formation, and use decreased gradually over time at all field types for the three shorebird species, excluding in the case of common greenshanks in seeded fields (Fig. 3). Excluding eight individuals at Julian date 150, large numbers of birds used the paddies in the early stages after formation, with a gradual decrease in use over time in the seeded field type in the case of the common greenshank (Fig. 1b).

**Table 2:** Results of the generalized linear mixed model examining the impact of field type and time since habitat formation on the abundance and distribution of shorebird species visiting the paddies. The sample sizes of black-tailded godwits, common greenshanks, and wood sandpipers were 809, 250, and 465, respectively.

Response variable	Estimated	S.E	*χ*^*2*^	*df*	*p*
**Black-tailed Godwit**					
Intercept	− 0.087	0.771	–	–	–
Period (Julian date)	21.934	0.220	13,945.190	1	< 0.0001
Time since formation	3.813	0.166	1,287.243	1	< 0.0001
Field type	1.496	0.148	475.989	2	< 0.0001
Water level	2.617	0.143	12.571	1	< 0.0001
Period $\times$ Field type	25.268	0.249	478.114	2	< 0.0001
**Common Greenshank**					
Intercept	− 15.087	4.719		–	–
Period (Julian date)	4.404	1.031	14.429	1	< 0.0001
Time since formation	0.543	0.111	0.076	1	0.783
Field type	0.297	0.172	17.271	2	< 0.0001
Water level	0.333	0.127	2.238	1	0.135
Period $\times$ Field type	4.501	1.098	17.343	2	< 0.0001
**Wood Sandpiper**					
Intercept	− 3.941	5.007	–	–	–
Period (Julian date)	8.957	0.828	101.600	1	< 0.0001
Time since formation	1.195	0.082	15.746	1	< 0.0001
Field type	0.425	0.179	6.941	2	< 0.05
Water level	1.036	0.092	12.098	1	< 0.001
Period $\times$ Field type	9.714	0.877	7.216	2	< 0.05

Each species was analyzed using density (individuals/paddy) as the response variable, field type (plowed, harrowed, or seeded) and time since habitat formation as the explanatory variables, and survey time and individual paddy as stochastic variables.

SE = standard error.

**Figure 4:**
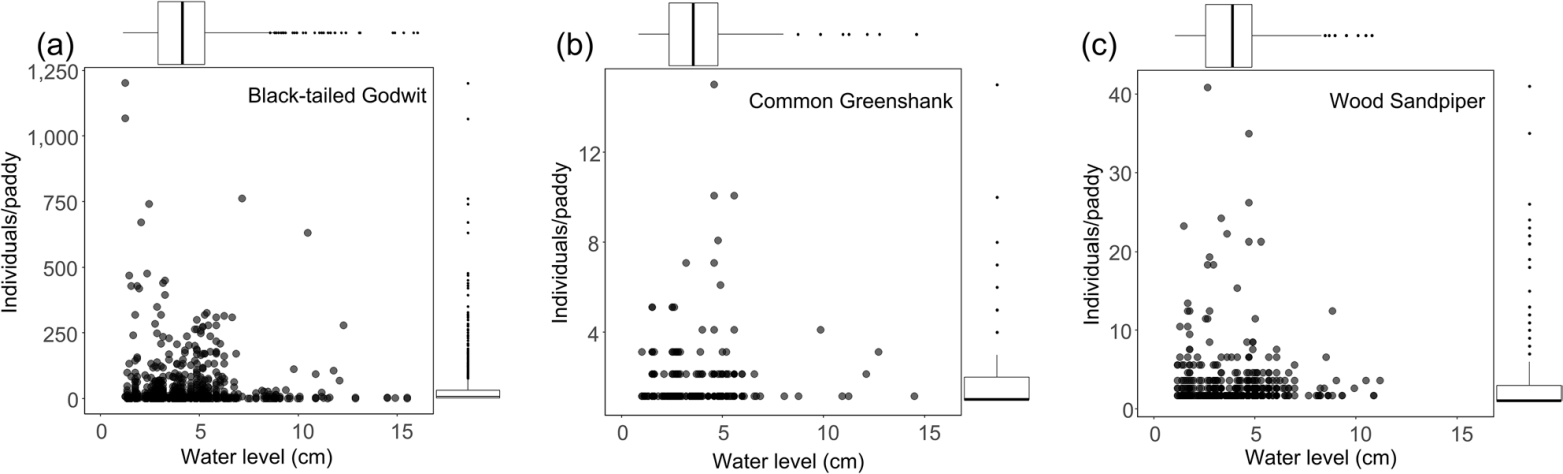
Scatterplot of water level and bird density (individuals/paddy) of shorebirds species. Each variable represents marginal box plots: (**a**) Black-tailed Godwit; (**b**) Common Greenshank; (**c**) Wood Sandpiper.

Water level also influenced density of black-tailed godwits and wood sandpipers significantly, based on the results of generalized linear mixed modelling (GLMM) (Table 2). The black-tailed godwits exploited a wider range of water levels than wood sandpipers (black-tailed godwit range: 1.29 – 23.34 water level; wood sandpipers range: 1.20–11.40 water level). The mean water levesl used by black-tailed godwits and wood sandpipers were 4.38 ± 2.14 and 4.20 ± 1.94, respectively (Fig. 4).

### Potential food sources

**Figure 5:**
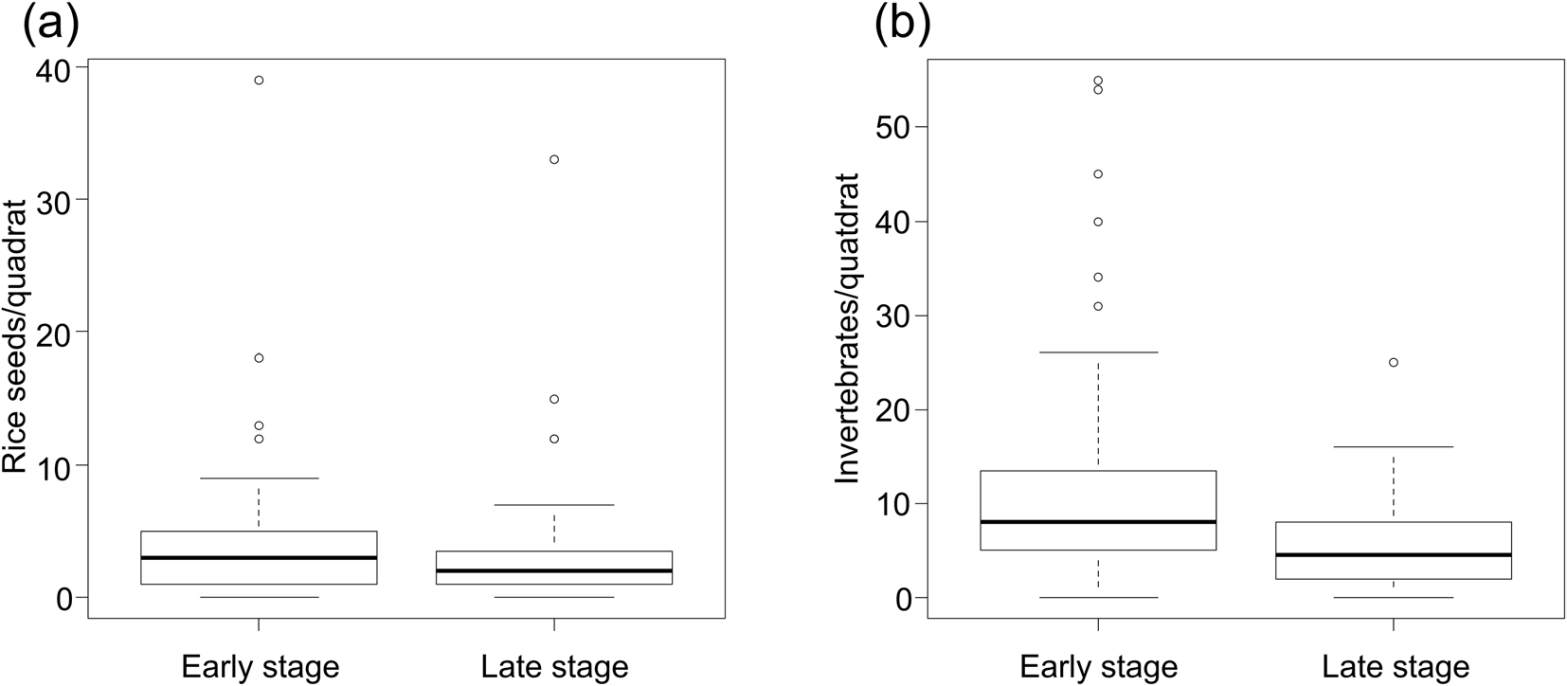
Box-and-whisker plots showing the abundance of potential food sources (rice seeds and benthic organisms) for shorebirds relative to time since habitat formation. The boxes represent the interquartile range for each food source, the horizontal line within the box is the median, and the whiskers show the range within the upper and lower limits. The circles represent outliers. The number of samples was 88 for the early stage (within 5 days of formation) and 80 for the late stage (after 10 days) habitats.

Total rice seed and benthic organism abundance were 659 (3.92 ± 0.38 individuals/paddy) and 1,394 (8.30 ± 0.67 individuals/paddy), respectively. The densities of rice seeds and benthic organisms (individuals/paddy), which are potential food sources for shorebirds, were not significantly associated with field type (Table 3), but were significantly and negatively associated with time since habitat formation (Table 3). Furthermore, number of individuals was higher in the early stage (within 5 days of formation) than in the late (after 10 days) stage (rice seeds: early = 4.89 ± 0.64; late = 2.86 ± 0.34; benthic organisms: early = 10.60 ± 1.13; late = 5.76 ± 4.92; Fig. 5).

**Table 3:** Results of the generalized linear mixed model examining the impact of field type and time since habitat formation on the abundance and distribution of potential food sources for shorebirds. The sample sizes of rice seeds and benthic organisms were both 56.

Response variable	Estimated	SE	*χ*^*2*^	*df*	*p*
**Rice seeds**					
Intercept	1.53	0.21	–	–	–
Field type	1.23	0.20	2.41	2	0.30
Time since formation	1.43	0.15	8.02	1	** < 0.01**
**Benthic organisms**					
Intercept	2.20	0.22	–	–	–
Field type	1.91	0.21	0.34	2	0.84
Time since formation	2.11	0.15	6.21	1	** < 0.05**

Each food source (rice seeds and benthic organisms) was analyzed using density (individuals per paddy) as the response variable, field type (plowed, harrowed, or seeded) and time since habitat formation as the explanatory variables, and survey time and individual paddy as stochastic variables.

SE = standard error.

## Discussion and conclusion

In the present study, we confirmed that shorebirds using paddy wetlands as stopovers in spring select paddies in different field types and with different water levels, as well as paddies that have existed for different periods of time, depending on the bird species. Our results suggest that field management in paddies influences habitat use by shorebirds. These results are consistent with those of many previous studies^17,34–37^. However, the previous studies were conducted during the non-growing season, whereas this study focused on paddy usage by shorebirds that occurred during the rice growing season. At the time of the arrival of shorebirds, the paddies are either being prepared for seeding or have recently been seeded. Therefore, most paddies are in the preparatory stages of rice cultivation at the time of arrival, although it is difficult to accurately determine the initial arrival time for shorebird species. During the period, field management activities gradually convert the paddy wetlands into states suitable for seeding. There are slight differences between paddies in the course of field management activities such as plowing, harrowing, and seeding, in addition to varying water levels. Consequently, it was found that rice paddy fields used by shorebirds are closely related by field management.

Field management activities such as plowing, harrowing, and seeding are conducted by machines and produce variations in physical structure of the soil condition. Although the three types of paddy habitat formed by field management had no effect on the amounts of food potentially available for shorebirds (see Table 3), the physical structure of each field type influences food availability for shorebirds^16^. Black-tailed godwits, common greenshanks, and wood sandpipers are more abundant in harrowed fields than in other habitat types. One of the most common foraging modes used by common greenshanks and wood sandpipers is pecking, that is using the bill tip and visual cues to capture prey at the sediment surface in rice fields^38^. Moreover, although black-tailed godwits mainly use the probing prey capture mode, they used the pecking mode more frequently in small flock sizes in rice fields^39^. Therefore, three shorebird species to foraging effectively at open ground^31,38^. Plowed and seeded field types may obscure prey from the three shorebird species. Conversely, harrowed fields are characterized by flat substrates, which provide ideal conditions for pecking prey capture modes of foraging^38^. Consequently, the three shorebird species exploited harrowed fields more than other habitat types.

Flooding in rice fields is also an important factor influencing how shorebirds use rice fields during spring migration. We could not observe the shorebirds in dried out rice fields; however, when the rice fields were flooded, the three shorebird species tended to concentrate on flooded rice fields, which has also been reported in other studies^40^. Other studies have also documented the use of flooded habitats by shorebirds in inland sites^41,42^. In addition, increase in water level by more than 10 cm is associated with a reduction in the number of the three shorebird species in rice fields. In the present study, black-tailed godwits and wood sandpipers mainly exploited rice paddy fields with shallow water levels (mean < 5 cm). Similarly, shorebirds reportedly prefer habitats with shallow water levels^43–46^, or habitats with levels < 10 cm^47–49^.

The time since habitat formation is an important factor in habitat use by shorebirds. Particularly in the early stage after habitat formation, paddies were used by large numbers of black-tailed godwits and wood sandpipers, but the number of birds continually decreased as the paddies entered the late stage. The birds’ preference for early-stage habitats is thought to be directly related to the amount of food. Early-stage habitats contained higher levels of rice seeds and benthic organisms than late-stage habitats (Fig. 3). Thus, food availability is higher in habitats shortly after field management such as plowing, harrowing, and seeding. Katayama et al*.*^50^ reported in 2015 that the use of agricultural machines increases physical disruption, which can affect the populations of mudfish, as well as amphibians, spiders, worms, and other invertebrates that might serve as food for birds. However, field management can also benefit birds. Tractors used for plowing are known to have a highly beneficial effect on food availability for cattle egrets, which are a representative paddy-using egret species^51,52^. Our study also showed that the use of agricultural machinery promoted the availability of potential food sources for shorebirds.

It is likely that common greenshanks showed no association with time since habitat formation because the number of birds per paddy was too low (see Table 3). A strong association with time since habitat formation was observed when the spatial scale was expanded to the size of the whole study area (approximately 460 ha; Spearman correlation *r* =  − 0.95; *p* < 0.0001; n = 10). Katayama et al*.*^53^ demonstrated in 2013 that the association between intermediate egrets and food in paddies needs to be investigated from various perspectives, depending on the spatial scale. Common greenshanks observed in paddies mostly use visual cues, moving around extensively as they look for food^29^. This feeding behavior means that when individual birds are more closely spaced, they have a mutually negative impact on food availability; accordingly, common greenshanks feed alone or form smaller, rather than larger, feeding groups. In the current study, the number of greenshanks per paddy ranged from 1 to 15, which was lower than that of black-tailed godwits or wood sandpipers (Table 1). The distance that greenshanks prefer to maintain between individuals for feeding may be larger than the size of each unit paddy, which is why there was no association between their presence and time since habitat formation at the spatial scale of unit paddies.

The current study demonstrated that dynamic rice-field management affect the feeding behavior of shorebirds. Field management in the spring may be closely related to the breeding of various shorebirds that use coastal inland rice paddies as migratory stopovers during their northward migration along the EAAF. In the paddies around the study area, the largest group of black-tailed godwits observed was over 1400 birds/ha (7300 individuals), whereas the largest groups of wood sandpipers and common greenshanks were 40 and 15 birds/ha, respectively. These numbers indicate the high dependence of this species on coastal inland paddy wetlands, supporting the observations of Choi et al.^19^. In particular, the black-tailed godwit, for which only 930 individuals were observed in important tidal flats in Korea, is typically observed in significant numbers in Korean rice paddies during its migration^54^. Furthermore, spring paddy management is particularly important for shorebirds because they only stop to rest and feed for a very short time, compared with other waterbirds such as egrets and ducks^29^. Therefore, it is clearly important to undertake effective conservation strategies for shorebirds that are distributed in the rice paddies during their northward migration along the EAAF.

In Korea, rice paddies are distributed broadly throughout the west coast, but shorebirds mainly use paddies in the central parts of Korea and rarely use paddies in the south. This usage pattern is because farms in the southern regions utilize two-crop farming in the spring, cultivating barley or onions before the rice-growing season; this technique leaves the soil dry, which makes it difficult for shorebirds to use as a feeding ground. Therefore, the central inland paddies might be critical for shorebirds on northward migrations along the EAAF. The central inland paddies used as migratory stopovers along the west coast need to fill with water and harrowed type—forming shallow wetlands—to be useful to the transiting birds. Furthermore, for the protection of the shorebirds using these paddies, systematic plans need to be established to manage the paddies during their brief visiting time. Even where field management activities establish land-use mosaic with different cultivation stages, the schedule should be managed to suit the timing of bird migration. We recommend that local farmers establish habitats and transition to other field types by performing cultivation around a 5–7-day cycle. In this period, the average number of individuals of black-tailed godwits and wood sandpipers is reduced by half, as the paddies enter the late stage. At each cultivation stage, the newly transitioned habitats are reset to the early stage in terms of desirability for the bird species. For this reason, the suggested 5–7-day period for cultivation may maximize food availability for shorebirds.

Rice fields located in the central part of Korea could have a major role in supporting the population of shorebird species along the EAAF during the northward migration period by supplementing their foraging opportunities. Our results suggest that field management would particularly be relevant for black-tailed godwits, common greenshanks, and wood sandpipers. Although we do not advocate for the development of new rice fields or other wetlands for the conservation of shorebirds, we advocate more appropriate management of existing rice fields facilities, since avian diversity in artificial wetlands is generally low relative to those in both restored and natural wetlands^55^. We propose that (1) flooding time, (2) shallow water level, (3) harrowed field type, and (4) 5–7-day management intervals at each paddy could better integrate shorebird conservation with rice field management. Such low-cost and simple procedures would not increase production costs or affect rice production, representing ‘win–win’ opportunities that could also enhance the environmental credentials of the product and ecosystem services^56^.

Our results suggest specific policies for the management of paddies that are used as stopover sites for shorebirds. However, in the current study, the relationship between shorebirds and their food sources was not fully elucidated. Therefore, future studies are required to identify the actual associations between shorebirds and food sources and to determine how field management affect the detailed distribution characteristics of potential food sources.

## Methods

### Study area

**Figure 6:**
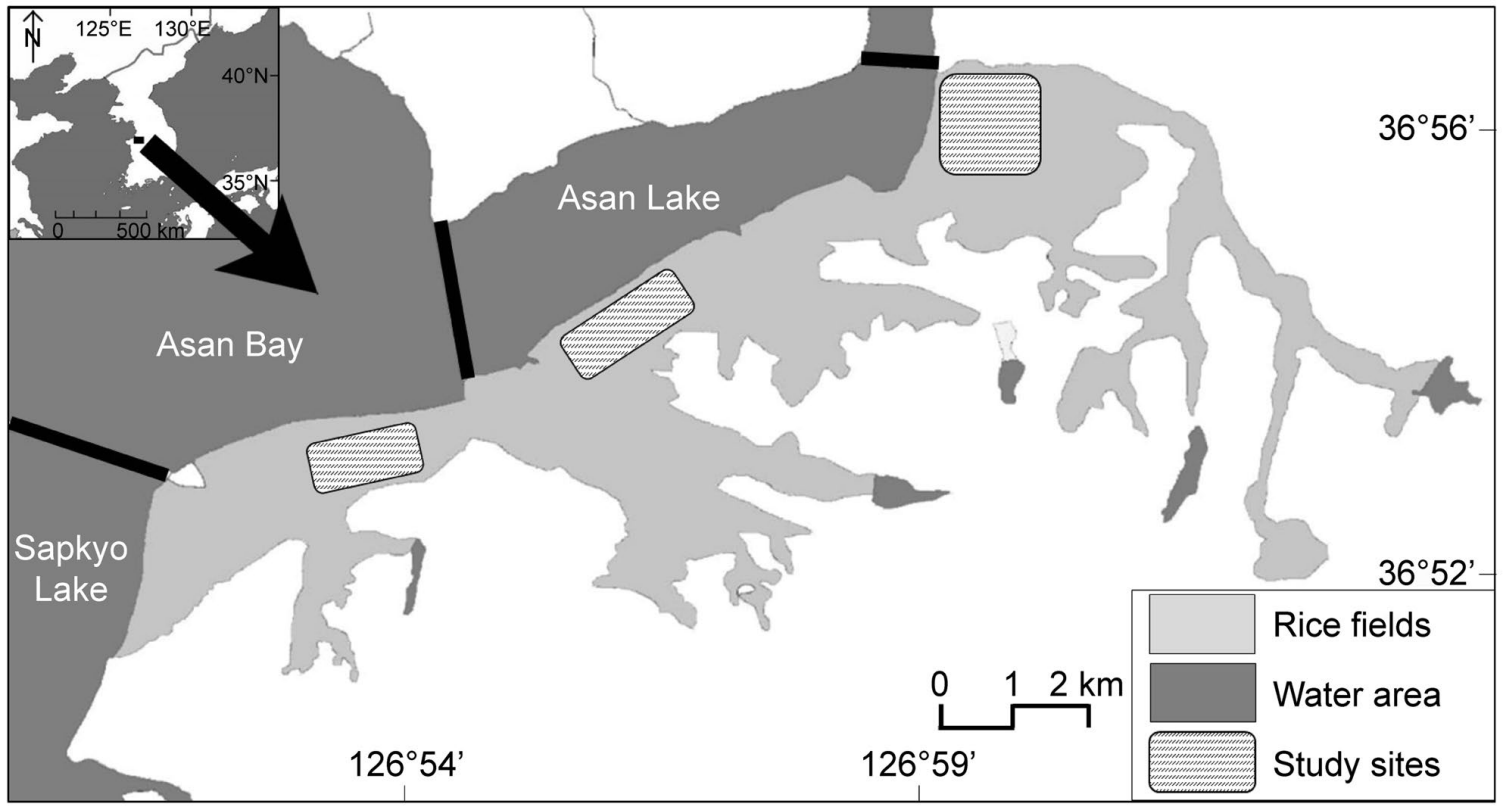
Map of the study area located in midwestern Korea, adjacent to Asan Lake and Asan Bay. The gray-shaded area is the overall rice-farming area, whereas the regions filled with dashed lines show the actual study sites. Created by Green Choi using Adobe Illustrator 2022.

The study was conducted in paddy wetlands located at the southern tip of Asan Bay and Asan Lake (36°54′ N, 126°56′ E; Fig. 6). This region is located within the EAAF and is used as a stopover by many long-distance migratory shorebirds. The paddy wetlands in the study area underwent readjustment as part of the Project for Comprehensive Development of Agriculture (CDA) in the 1970s, during which the previously irregular and fragmented farmland was standardized, farm roads were constructed to facilitate the passage of agricultural machines, and traditional-style drainage systems were converted to modern water supply/drainage routes. The average size of the standardized unit paddies (separated by ridges) was 0.45 ± 0.07 ha (range: 033–0.66 ha); the study area consisted of 1,022 unit paddies.

### Bird and habitat surveys

We checked shorebird occurrence and paddy type in all the paddies (1,022 units) at 2–3-day intervals from April 1 (Julian date 091) to June 10 (Julian date 161), 2014 during spring migration (38 times). Bird surveys were conducted 18 times during these periods, at 2–3-day intervals, over a period of 40 days between April 21 (Julian date 111) and May 30 (Julian date 150), 2014, when shorebirds resided in the study area. The surveys were conducted between 6:00 am and 12:00 pm; observers walking on the farm roads used a telescope and binoculars to identify the species and numbers of shorebirds per unit paddy. All paddies were directly adjacent to farm roads throughout the CDA project. Surveys were not conducted on rainy days, minimizing the effect of weather on the data.

**Figure 7:**
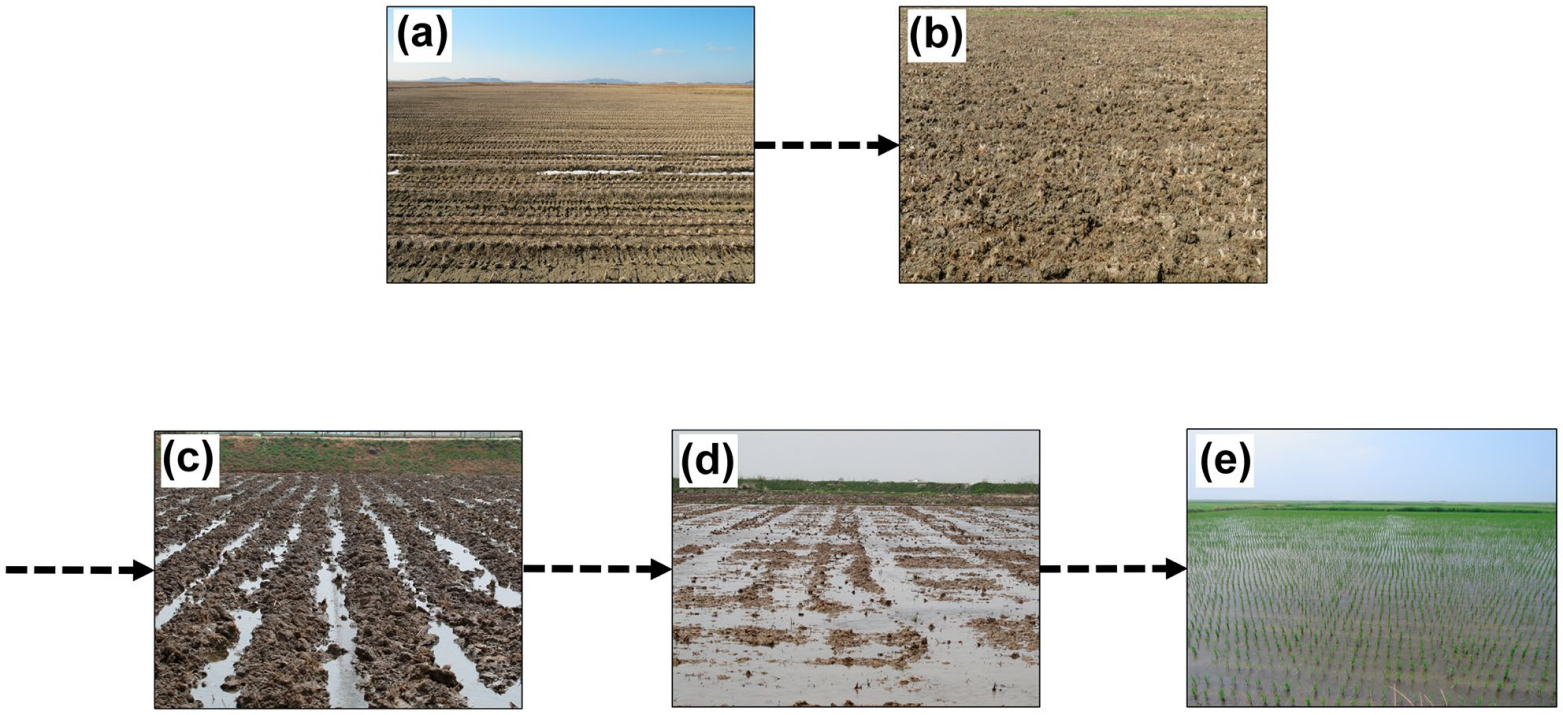
Progress of major changes in field types. Two types of paddy habitats observed at the study site before shorebird occurrence: (**a**) post-harvest and (**b**) plowed before filling with water. Three types of paddy habitats observed at the study site during the period of northward shorebird migration: (**c**) plowed paddy, (**d**) harrowed paddy, and (**e**) seeded paddy.

Formation of habitats was considered to occur when the paddy was plowed, harrowed, or seeded, and habitats formed during the survey period were divided into three categories based on those three types of field management. Although all of the paddies were submerged, they were differentiated by the state of the soil surface and the presence or absence of seeds. The field types (Fig. 7) were as follows: (1) watered paddy after plowing, (2) watered paddy after harrowing, and (3) seeded paddy. At the beginning of the study period (April 1), the paddies in the study area were in a post-harvest or post-plowing state, but before filling with water. Shorebirds did not use the paddies in this state. Generally, field types changed in the order of post-harvest, plowing before filling with water, watered paddy after plowing, watered paddy after harrowing, and seeded paddy. Some habitats, however, changed directly from post-harvest to watered paddy after plowing or from plowing without water to watered paddy after harrowing (see Fig. 7). On April 21, shorebirds were first observed and field types were starting to fill with water for the first time. To identify the time since habitat formation, a unique identifying number was assigned to each unit paddy, and changes in the state of each paddy were checked at 2–3-day intervals. We measured water level (cm) at feeding points where bird activity was observed, for example, based on footprints or probed (or pecked) holes in shorebird-foraged paddies. Water level was measured 15 times in each paddy and the mean represented the level in each paddy.

### Food source survey

From April 22 to May 30, 2014, we examined how paddy type and time since habitat formation affected the abundance of rice seeds and benthic organisms, which are potential food sources for shorebirds. Quantitative surveys were conducted using a 20 cm × 20 cm quadrant for collection. From each paddy, four soil samples were collected at a depth of 5 cm; the samples were passed through a 500-µm sieve on-site, fixed in 70% alcohol, and then transported to the laboratory. Collected food sources were divided into benthic organisms and rice seeds, and the quantity of each was calculated. In addition, samples were collected from each of the three field types to analyze abundance by time (early and late stage). During surveys in the filed, the time since habitat formation was determined to be 12–15 days; therefore, the “early” stage was defined as within 5 days of habitat formation, and the “late” stage was defined as at least 10 days after formation. Surveys were conducted 56 times in each of the paddy types, including plowed paddies (early: 32 times, late: 24 times), harrowed paddies (early: 28 times, late: 28 times), and seeded paddies (early: 28 times, late: 28 times). For all collections, we randomly selected paddies in which shorebirds and other waterbirds had not yet been observed. Only one sampling event was carried out in one paddy. The total sample size was 168 (56 samples in each field type).

### Statistical analysis

A GLMM (Poisson distribution and log link) was used to confirm the effects of period (Julian date), time since habitat formation, field type, water level, and interaction between period and field type on shorebird number. GLMM (Poisson distribution and log link) was also used to confirm the suitable field type and time since habitat formation with regard to abundance of potential food sources. For each species of bird observed, the number of individuals per paddy and the abundance of food sources were designated as response variables, whereas time (Julian date), time since habitat formation, field type, water level, and interaction of time and field type were designated as explanatory variables. Individual paddies were designated as stochastic variables. The predicted value for change in the number of individuals using each paddy, by species and time since formation, was expressed as a marginal effect. Marginal effect is a good method for estimating change because it calculates the marginal probability of the response variable (number of individuals using each paddy) for a unit change (2–3-day intervals) in an explanatory variable (time since habitat formation). R version 3.2.1 was used for all statistical analyses^57^. The lme4 packages were used for GLMM^58^, and the sjPlot package was used to display marginal effects^59^.

## Data Availability

The datasets used and/or analysed during the current study are available from the corresponding author on reasonable request.
